# The role of cannabinoid receptor 2 in oligo/poly-articular juvenile idiopathic arthritis

**DOI:** 10.1186/1546-0096-12-S1-P34

**Published:** 2014-09-17

**Authors:** Maria F Gicchino, Giulia Bellini, Laura Perrone, Emanuele Miraglia del Giudice, Francesca Rossi, Maria Alessio, Anna Grandone, Alma Nunzia Olivieri

**Affiliations:** 1 Department of Women,Child and General and Specialistic Surgery, Naples, Italy; 2 Department of Experimental Medicine, Second University of Naples, Naples, Italy; 3 Department of Translational Medical Sciences, University of Naples Federico II, Naples, Italy

## Introduction

Juvenile Idiopathic Arthritis(JIA) is an inflammatory chronic disease concerning joints and others structures. According to International League of Association for Rheumatology (ILAR) seven subtypes of arthritis can be defined in relation with the number of joints and the extra-articular involvement occurring in the first six months of disease. Although JIA pathogenesis is not completely clear is known that T-cell activation is a feature of oligoarticular and polyarticular JIA. The endocannabinoid system is involved in immune regulation by reducing cells activation, modulating Th1 and Th2 balance, inhibiting proinflammatory cytokine production. T-cell and other cellular components of immune system express cannabinoid receptor 1 and 2 (CB1-CB2).

## Objectives

It has been demonstrated that a CB2 common variant, glutamine-arginine substitution Q63R, differently modulated the EC-induced inhibition of T-cells proliferation. T lymphocytes from RR homozygotes had two fold reduction of EC-induced inhibition of proliferation compared to those from QQ homozygotes, suggesting this CB2 variation as a risk factor for autoimmune diseases. The aim of this study is to investigate whether the functional variant Q63R of CB2 is associated with the susceptibility to oligo/polyarticular JIA and with its clinical features.

## Methods

This study includes 171 children suffering from JIA(124 females;47 males) genotyped for the CNR2 rs35761398 variant causing the substitution Q63R. JIA diagnosis was made according to ILAR criteria and treatment was assigned with recommendations of the American College of Rheumatology. For each patient we evaluated number of affected joints, age of onset , comorbidities, presence of autoimmune diseases associated and relapses. The presence of uveitis was considered a comorbidity. Celiac disease, thyroiditis and diabetes mellitus were considered autoimmune disease associated. According to Wallace criteria any symptom appearing after six months from remission was considered as relapse of arthritis. 600 healthy children previously genotyped for the CB2 Q63R functional variant were used as controls. P values less than 0.05 were considered significant. The odds ratio was evaluated considering the presence of at least one single risk allele (heterozygous QR plus homozygous RR vs homozygous QQ) or two( homozygous RR vs homozygous QQ and heterozygous QR).

## Results

We genotyped 105 oligoarticular and 66 polyarticular JIA affected children for the CNR2 rs35761398 variant. We analyzed the same variant in 600 healthy controls. The allelic frequencies and the genotype distributions of CB2 polymorphism showed a significant difference between patients and controls (p=0,03;p=0,0001 respectively). The relative odds ratios (OR) revealed a double risk for developing oligo/polyarticular JIA in patients with at least one R63 allele, respect to QQ homozygotes( OR=1.905; C:I 95% 1.06-3.47;p= 0,02). We found a significant association of the CB2 Q63R variant with relapse of arthritis (p=10^-4^).Finally we found a significant difference in the genotype distribution of the CB2 Q63R variant between oligoarticular compared to poliarticular subtype: the percentage of RR homozygotes was higher in patients with polyarticular JIA (52% vs 42%; p=0.04)

## Conclusion

Data found in this study indicate the CB2 receptor as a possible molecular determinant contributing to susceptibility to oligo/polyarticular JIA and to its clinical course.

## Disclosure of interest

None declared.

